# Spike protein multiorgan tropism suppressed by antibodies targeting SARS-CoV-2

**DOI:** 10.1038/s42003-021-02856-x

**Published:** 2021-11-22

**Authors:** Molly Brady, Conor McQuaid, Alexander Solorzano, Angelique Johnson, Abigail Combs, Chethana Venkatraman, Akib Rahman, Hannah Leyva, Wing-Chi Edmund Kwok, Ronald W. Wood, Rashid Deane

**Affiliations:** 1 Department of Neuroscience, Del Monte Institute of Neuroscience, University of Rochester, URMC, 601 Elmwood Avenue, Rochester, NY 14642 USA; 2 Department of Imaging Sciences, University of Rochester, URMC, 601 Elmwood Avenue, Rochester, NY 14642 USA; 3 Departments of Obstetrics and Gynecology, University of Rochester, URMC, 601 Elmwood Avenue, Rochester, NY 14642 USA; 4 Department of Urology, University of Rochester, URMC, 601 Elmwood Avenue, Rochester, NY 14642 USA

**Keywords:** Blood-brain barrier, Viral infection

## Abstract

While there is SARS-CoV-2 multiorgan tropism in severely infected COVID-19 patients, it’s unclear if this occurs in healthy young individuals. In addition, for antibodies that target the spike protein (SP), it’s unclear if these reduce SARS-CoV-2/SP multiorgan tropism equally. We used fluorescently labeled SP-NIRF to study viral behavior, using an in vivo dynamic imaging system and ex in vivo tissue analysis, in young mice. We found a SP body-wide biodistribution followed by a slow regional elimination, except for the liver, which showed an accumulation. SP uptake was highest for the lungs, and this was followed by kidney, heart and liver, but, unlike the choroid plexus, it was not detected in the brain parenchyma or CSF. Thus, the brain vascular barriers were effective in restricting the entry of SP into brain parenchyma in young healthy mice. While both anti-ACE2 and anti-SP antibodies suppressed SP biodistribution and organ uptake, anti-SP antibody was more effective. By extension, our data support the efficacy of these antibodies on SARS-CoV-2 multiorgan tropism, which could determine COVID-19 organ-specific outcomes.

## Abstract (web summary)

Brady et al. demonstrate that spike protein-targeting antibodies reduce SARS-CoV-2 biodistribution. This highlights potential avenues for vaccine development.

## Introduction

The recent pandemic is caused by a coronavirus (CoV) called severe acute respiratory syndrome coronavirus 2 (SARS-CoV-2), which elicits SARS, an infectious disease, coronavirus disease 2019 (COVID-19)^1^. The virus’ main route of entry into the body is by inhalation of droplets; thus, the disease is manifested as respiratory dysfunction leading to pneumonia in severe cases^2–10^. The virus enters host cells by interacting with facilitators, mainly angiotensin converting enzyme 2 receptor (ACE2), which is widely distributed in many tissues^10–19^. The spike protein (SP) on the viral lipid membrane (a crown-like appearance) avidly binds with membrane-bound ACE2^20^. However, many non-respiratory organs are also affected, such as the heart^21–23^, kidneys^24,25^, liver^26–29^, and brain^3,30–35^, which can lead to multiorgan failure in severe cases of susceptible individuals. However, most of the COVID-19 cases are asymptomatic, mild, or moderate^5,36,37^. It is unclear whether SARS-CoV-2 is distributed equally to all organs in these cases. In addition, for antibody therapies, such as anti-SP, and vaccines that target the SP, it is unclear whether these reduce SARS-CoV-2 multiorgan biodistribution equally. In vaccines, such as mRNA vaccines, the translated SP is released into interstitial fluid/blood, distributed to many organs and triggers an immune response. Thus, we studied the biodistribution of intravenously injected SP and tested the effect of anti-ACE2 and anti-SP antibody on SP regional biodistribution and organ uptake using an external in vivo dynamic imaging system and ex in vivo tissue analysis.

Herein, we show that SP had a body-wide biodistribution, slow regional elimination, except for the liver, which showed an accumulation, and differential organ uptake. SP uptake was highest for the lungs and this was followed by the kidney, heart, and liver, but lowest in the brain. SP was present in the choroid plexus (CP) but there were no detectable SP levels in the cerebrospinal fluid (CSF). Thus, the brain vascular barriers were effective in restricting the entry of a viral protein (SP) into brain parenchyma and CSF in young healthy mice. Also, SP was present in the salivary glands, duodenum, and spleen. Although both anti-ACE2 and anti-SP antibodies suppressed SP regional biodistribution and organ uptake, anti-SP antibody was more effective. The data suggested that these antibodies, especially anti-SP antibody, can effectively reduce the SP biodistribution and multiorgan tropism, and thus may contribute to the efficacy of these therapies or vaccines.

## Results

### SP is widely distributed and slowly eliminated

**Fig. 1:**
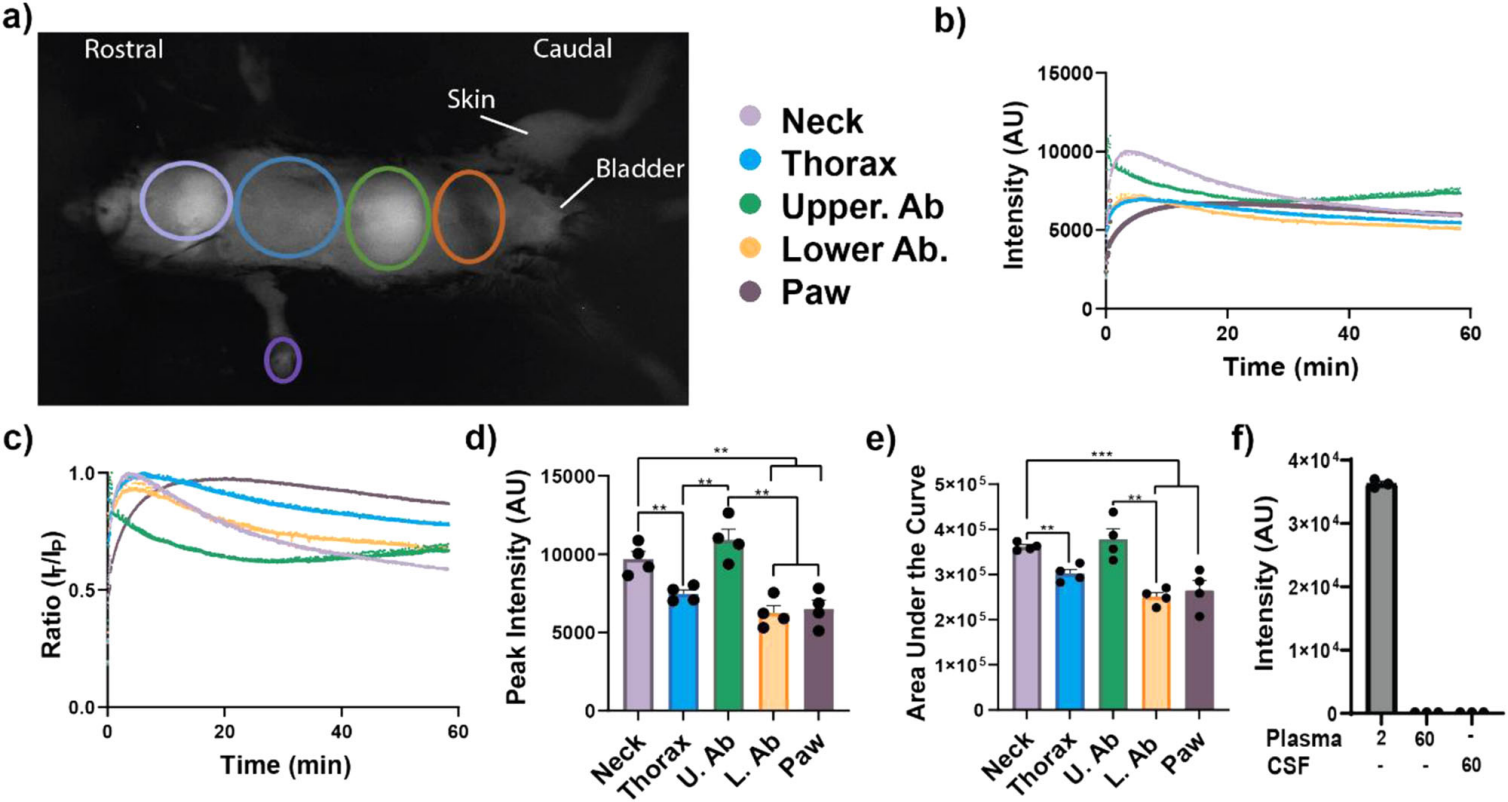
Body-wide biodistribution and slow elimination of a SP in mice. **a** External in vivo dynamic SP-NIRF image of a mouse after its intravenous injection (after a few minutes). Regions of interest (ROIs) selected for the analysis. **b** Representative SP-NIRF intensity-time profile for each ROI over 60 min. **c** Standardization of data by dividing intensities at each time point by the peak intensity (*I*_T_/*I*_P_ ratio). **d** Peak intensity for the ROIs. **e** Area under the curve (AUC) for the ROIs. Values are mean ± SEM, *N* = 4 young male mice (2–3 months old). **f** Plasma intensities at 2 and at 60 min, and CSF levels at 60 min. Values are mean ± SEM, *N* = 3 young male mice. AU (arbitrary units).

First, the biodistribution pattern of a SARS-CoV-2 SP (SP-NIRF), after intravenous injections, in 2- to 3-month-old male adult mice was established. The SP-NIRF signal was acquired for the whole mouse in its supine position to simultaneously observe regions that are likely affected by the virus, using an external imaging system (Supplementary Fig. 1a). Thus, for this analysis, the regions of interest (ROIs) were the neck, thorax, upper abdomen, lower abdomen, and paw based on preliminary imaging (Fig. 1a). We confirmed that NIRF signal can be detected under the rib cage (Supplementary Fig. 1b), as we reported for the brain^38^. Following the injection, SP-NIRF signal was increased to a peak then gradually decreased in all these ROIs, except the upper abdomen, which slightly increased after a trough at about 30 min (Fig. 1b). Although there was signal on the whole mouse, including the skin, it was most pronounced for the neck (mainly salivary glands) and the upper abdomen (mainly the liver). To correct for possible experimental variations, the data were standardized to the peak signal and the same patterns for the signal profile were observed at each ROI (Fig. 1c).

The peak SP-NIRF signals were highest for the neck and upper abdomen, which was 1.3- to 1.9-fold greater than that for the thorax, lower abdomen, and paw, but there was no significant difference between these regions (Fig. 1d). Although the area under the curve (AUC), the overall regional SP-NIRF exposure, was also similar for the upper abdomen and neck, these were greater by 1.30- to 1.8-fold compared to the thorax, lower abdomen, and paw. However, there was no significant differences between the AUC for the thorax, lower abdomen, and paw (Fig. 1e).

The elimination rate constants were no significant for the neck, thorax, and lower abdomen, which had elimination phases (Supplementary Fig. 1c). For the upper abdomen, after its distribution, the rate of SP-NIRF accumulation was determined from the rising phase of the intensity-time profile, using linear regression analysis (Supplementary Fig. 2d). SP-NIRF signal was detected in the bladder but this was variable, perhaps, due to changes in urination, as this was not controlled (Supplementary Fig. 1e). SP-NIRF plasma and CSF levels were not detectable at 60 min (Fig. 1f), whereas the ROI signals were higher, suggesting that there was retention within these regions.

In summary, these data show that SP-NIRF was widely distributed within the body of young mice, and taken up in many organs, especially in the upper abdominal (mainly liver) and neck (mainly salivary glands) regions. There was an elimination phase for the neck, thorax, and lower abdominal regions, whereas for the upper abdominal region there was accumulation of SP-NIRF after its distribution, instead of elimination. Peak levels were highest for the neck and upper abdominal regions, which may indicate greater uptake even though there was an elimination phase for the neck region but an accumulation phase for the upper abdominal region. SP-NIRF signals in the paw increased exponentially to a plateau and was almost constant thereafter. By extension, these data suggest that there will be differential multiorgan tropism of the virus in young mice, which could lead to organ dysfunction/failure in susceptible individuals, assuming SP mimics the viral distribution, as this is due mainly to SP–host cells interaction.

### SP uptake suppressed by anti-ACE2 and anti-SP antibodies

ACE2 receptor is widely distributed in the vasculature and in tissues, including the lungs, kidneys, heart, and possibly the cerebrovasculature^10–12,14,19,32,39,40^. SP interaction with ACE2 receptor would indicate potential viral uptake regions and anti-ACE2 antibody will decrease viral–host cell interaction. Anti-SP antibody, as used in passive immunity, generated in active immunity, and produced by vaccines, will interact with the SP on SARS-CoV-2 to eliminate the virus and also reduce viral–host cell interaction. For some of the vaccines, the SP produced by the vaccine will be released from cells and distributed in the body before it can mount an immune response. Anti-SP antibody in the circulation can then interact with the SP on the virus. Thus, we tested the effect of anti-SP antibody and anti-ACE2 on the regional biodistribution, elimination kinetics, and organ uptake of an SP to establish whether there are differential effects. We confirm that the anti-ACE2 antibody interacts with mouse ACE2 (Supplementary Fig. 2a). Also, we show that SP-555 interacts with mouse and human ACE2 at SP concentrations between 0.01 and 1.0 μg ml^−1^ (Supplementary Fig. 2b). At lower SP-555 concentrations there were binding to human ACE2 (higher affinity), whereas there was little binding to mouse ACE2. In our IV studies, we administered 2.05 μg ml^−1^ of SP-555, and assuming that plasma volume is ~1 ml, the plasma SP concentration should be ~2.0 μg ml^−1^, at the peak, which may contribute to the results.

**Fig. 2:**
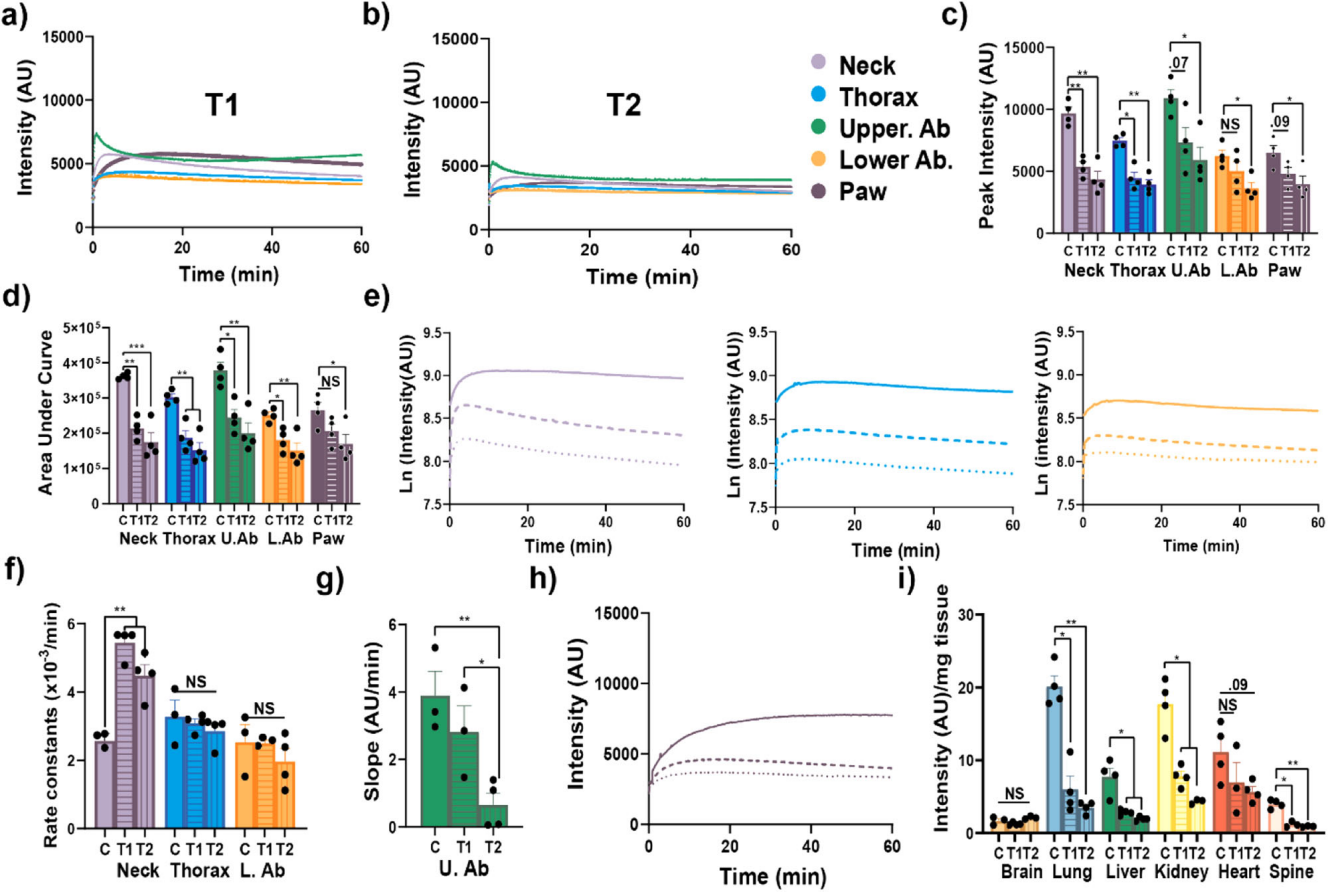
Anti-ACE2 and anti-SP antibodies reduced SP biodistribution. **a**, **b** Representative SP-NIRF intensity-time profile at the ROIs for T1 (anti-ACE2 antibody) and T2 (anti-SP antibody) in 2- to 3-month-old mice after its intravenous injection. **c** Peak intensity for the ROIs. **d** Area under the curve (AUC) for the ROIs. **e** Semi-Ln plots for the intensity-time profile of the neck, thorax, and lower abdomen ROIs (continuous line (control); dashed line (anti-ACE2 antibody) and dotted line (anti-SP antibody). **f** Elimination rate constants for the neck, thorax, and lower abdomen. **g** Slopes for the accumulation phase for the upper abdomen. **h** Intensity-time profile of the paw. **i** Organ SP-NIRF intensities/mg wet weight at 60 min. Values are mean ± SEM, *N* = 4 young male mice. AU (arbitrary units).

In separate groups of mice, anti-ACE2 antibody (T1, 10 μg) or anti-SP antibody (T2, 10 μg) was injected 15 min prior to the SP-NRF injections. Although the general pattern of the ROIs intensity profile curves was similar, the values were lower than that for the young control mice (Fig. 2a, b, Supplementary Fig. 2b, c, and Fig. 1b). The peak SP-NIRF signals were significantly higher in the control mice compared to the T1-treated mice, by 1.8-fold for the neck and thoracic regions but not significantly for the upper abdomen, lower abdomen, and the paw (Fig. 2c). In contrast, for T2 treatment it was higher for the controls by two- to threefold for all ROIs (Fig. 2c). The AUC was significantly greater in the control mice compared to the T1- or T2-treated mice by 1.8- to 2.5-fold in all the ROIs, except for T1 and the paw (Fig. 2d). The intensities were transformed to the Ln scale and the elimination rate constants determined from the disappearance phase of the graph (Fig. 2e). T1 and T2 increased the elimination rate constants for the neck by 2.0- to 2.5-fold but not for the thorax and upper lower abdomen compared to controls (Fig. 2f). For the upper abdomen, the rate of SP-NIRF accumulation after its distribution was reduced by 5.5-fold with T2 but not by T1 compared to controls (Fig. 2g). Also, T1 and T2 reduced SP-NIRF distribution (AUC) for the paw (Fig. 2h).

Collectively, these data show that both T1 and T2 reduced the biodistribution to the ROIs, with T2 (anti-SP antibody) more effective, in general. Interestingly, for the neck region (mainly the salivary glands, but also include the pharynx and larynx) T2 was effective in increasing the removal of SP-NIRF (higher elimination rate constant). Similarly, T2 was effective in reducing the accumulation phase of the upper abdomen (mainly the liver). For the paw T2 reduced SP-NIRF peak intensity and the AUC. In contrast, for the thorax and lower abdomen, there are many potential sources of the SP-NIRF signals, including lungs and heart for the thorax, and intestines and kidneys for the lower abdomen. Thus, by extension, therapies targeting ACE2 and the SP, and especially vaccines targeting SP are effective in suppressing the biodistribution of the virus to organs in this model.

### Ex vivo organ SP distributions

To better understand which organ was associated with the SP-NIRF, at the end of the experiment, these (brain, lungs, liver, kidneys, spleen, intestine, and spine) were removed, washed in cold phosphate-buffered saline (PBS), and imaged using the same parameters as in the in vivo imaging. There were no significant differences in the intensities between the dorsal and ventral surfaces of the brain even though there are more visible surface blood vessels on the ventral surface (Supplementary Fig. 2d). As these organs vary in weight, signal intensities were standardized per unit wet weight. SP-NIRF uptake into the lungs, kidney, and heart was 8.0- to 12-fold greater than that of brain (Supplementary Fig. 2e). The brain had the lowest SP-NIRF uptake, whereas the lungs had the highest. SP-NIRF signals for the spleen was low, but could contributed to the upper abdominal ROI (Supplementary Fig. 2f, g). Likewise, the intestine contributed to the SP-NIRF signal for the lower abdomen. Interestingly, SP-NIRF signal for the small intestine was mainly detected in the duodenum (Supplementary Fig. 2h). After 60 min, there was very low SP-NIRF levels for the salivary glands (Supplementary Fig. 2i). Other tissues may contribute to signal in the neck region, such as the trachea, tongue, and larynx and pharynx. T1 and T2 reduced the SP-NIRF uptake by the liver, lungs, and kidney by four- to sevenfold, but not for the heart and brain compared to control young mice (Fig. 2i). T1 and T2 had no significant effect on the SP-NIRF uptake by the brain. These data show that anti-SP antibodies can effectively reduce SP multiorgan biodistribution and, thus, by extension should reduce the virus effects on these regions, in this model.

### Ex vivo analysis of tissue sections

**Fig. 3:**
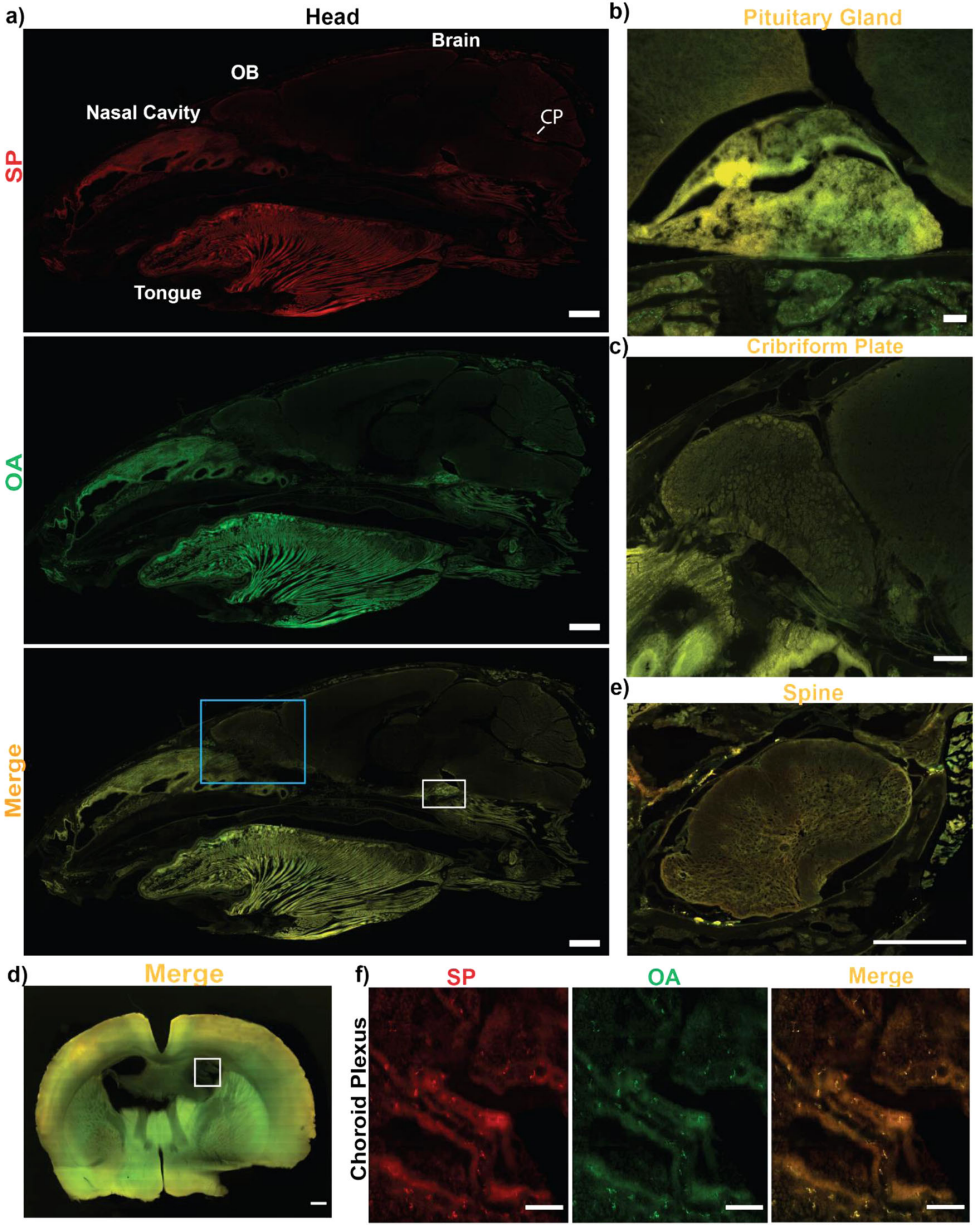
SP is not present in brain parenchyma. **a** Representative sagittal section of the head for a young mouse showing distribution of SP-555, OA-488, and merged images. OB (olfactory bulb). CP (choroid plexus). **b** Magnified region (pituitary gland) of the white box in **a**. **c** Magnified region (nasal/cribriform plate) of the blue box in **a**. **d** Representative coronal sections of the merged images of SP-555 and OA-488. **e** Magnified region (choroid plexus) of the box in **d**. **f** Merged image of the spinal cord showing the present of both tracers. Scale bars: **a** (1 mm); **b**–**d** (100 μm); **e** (0.5 mm). Values are mean ± SEM, *N* = 3. Images enhanced to better show details, but not used for analysis.

To establish whether the SP was taken up within the organs we analyzed SP-555 uptake and compared it to a reference protein of similar molecular weight (ovalbumin, OA-488). Sagittal sections of the head show that most of the SP-555 and OA-488 were present in peripheral tissues, with limited entry, if any, into the brain parenchyma (Fig. 3a) compared to that of brain sections from non-injected mice (Supplementary Fig. 3a, b). However, there were SP and OA signals in the region associated with pituitary gland, which lacks a blood–brain barrier (BBB), and the CP, but not across the cribriform plate (Fig. 3a–c). Coronal brain sections confirmed the absence of detectible SP-555 signal in brain parenchyma, although there were signals for both SP-555 and OA-488 associated with the CP compared to samples from non-injected mice (Fig. 3d and Supplementary Figs. 3b and 4a). There was no significant association of SP with the olfactory bulb, blood vessels, or neurons (Supplementary Fig. 4b–d). In contrast, the spinal cord had detectible signals for both SP-555 and OV-488, compared to samples from non-injected mice (Fig. 3f and Supplementary Figs. 3c and 4e, f), possibly due to a greater permeability of the spinal cord capillaries than that of brain^41^. Thus, the low brain SP-NIRF levels could be due mainly to the CP and possibly areas lacking BBB, such as the pituitary gland. Thus, the central nervous system (CNS) vascular barriers seem to be effective in limiting SP entry into brain parenchyma, in young healthy brains.

**Fig. 4:**
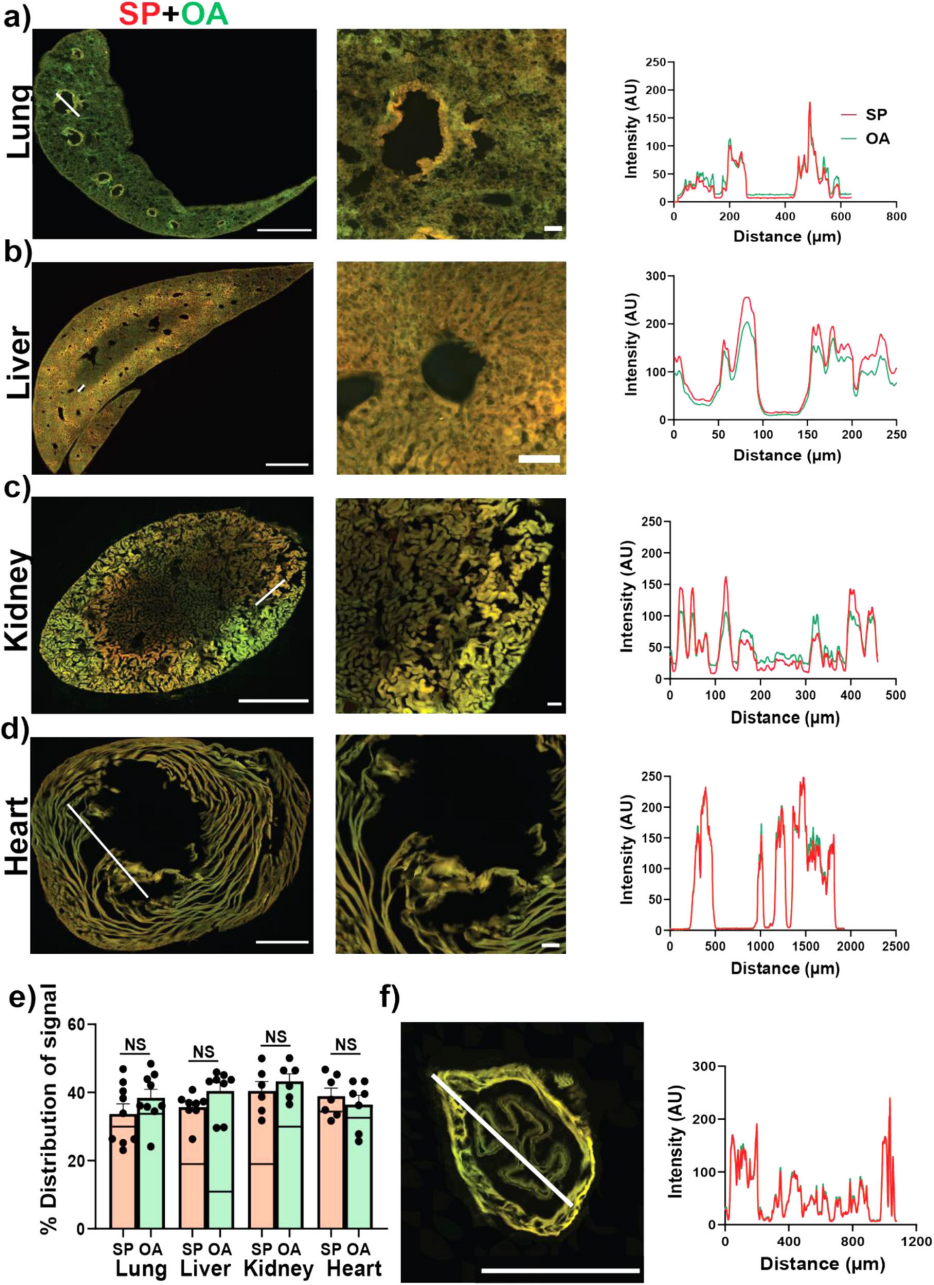
SP-555 peripheral tissues biodistribution. **a**–**d** Representative merged images of SP-555 and OA-488 with magnified images and intensity plots at the respective white lines. **e** Quantification of SP-555 and OA-488 distribution area for the whole section with the auto fluorescence levels (lines) from the non-injected mice. **f** Merged image of a primary bronchus with intensity plot. Scale bars: **a**–**d** and **e** (1 mm). Values are mean ± SEM, *N* = 3–4 mice. Images not enhanced.

For the peripheral tissues, there were significant distribution of SP-555 and OA-488 in whole-section analysis for the lungs, liver, kidney, and heart (Fig. 4a–e and Supplementary Fig. 5a–d) compared to the non-injected mice (Supplementary Fig. 5e–h). However, the level of auto fluorescence was high in these tissues, which range from almost all of the signal (as seen in the heart) to about 40% for 555 nm signals. Thus, in whole section, the analysis of fluorescence level at these wavelengths (555 and 488 nm) may not be as relevant in these studies. However, intensity plots show that there were higher levels locally, such as that associated with the airways (bronchioles), liver lobule, kidney tubules, and heart tissues (Fig. 4a–d). The intensity plots of the primary bronchus showed similar distribution as the smaller bronchiole (Fig. 4f). Collectively, these data indicate that the CNS vascular barriers restrict the entry of SF-555 and OA-488 but not for peripheral tissues (such as lungs, liver, kidneys, and heart). The biodistribution and tissue uptake may be due to ACE2 receptors in these tissues, and to other facilitators, such nonspecific entry mechanisms. Liver maybe involved in the degradation/elimination of proteins, such as SP. Both of these molecules will be filtered and excreted by the kidneys. SP may be reabsorbed by the kidney tubules, including the proximal tubules^24,25^.

### SP-555 uptake by the isolated CP

**Fig. 5:**
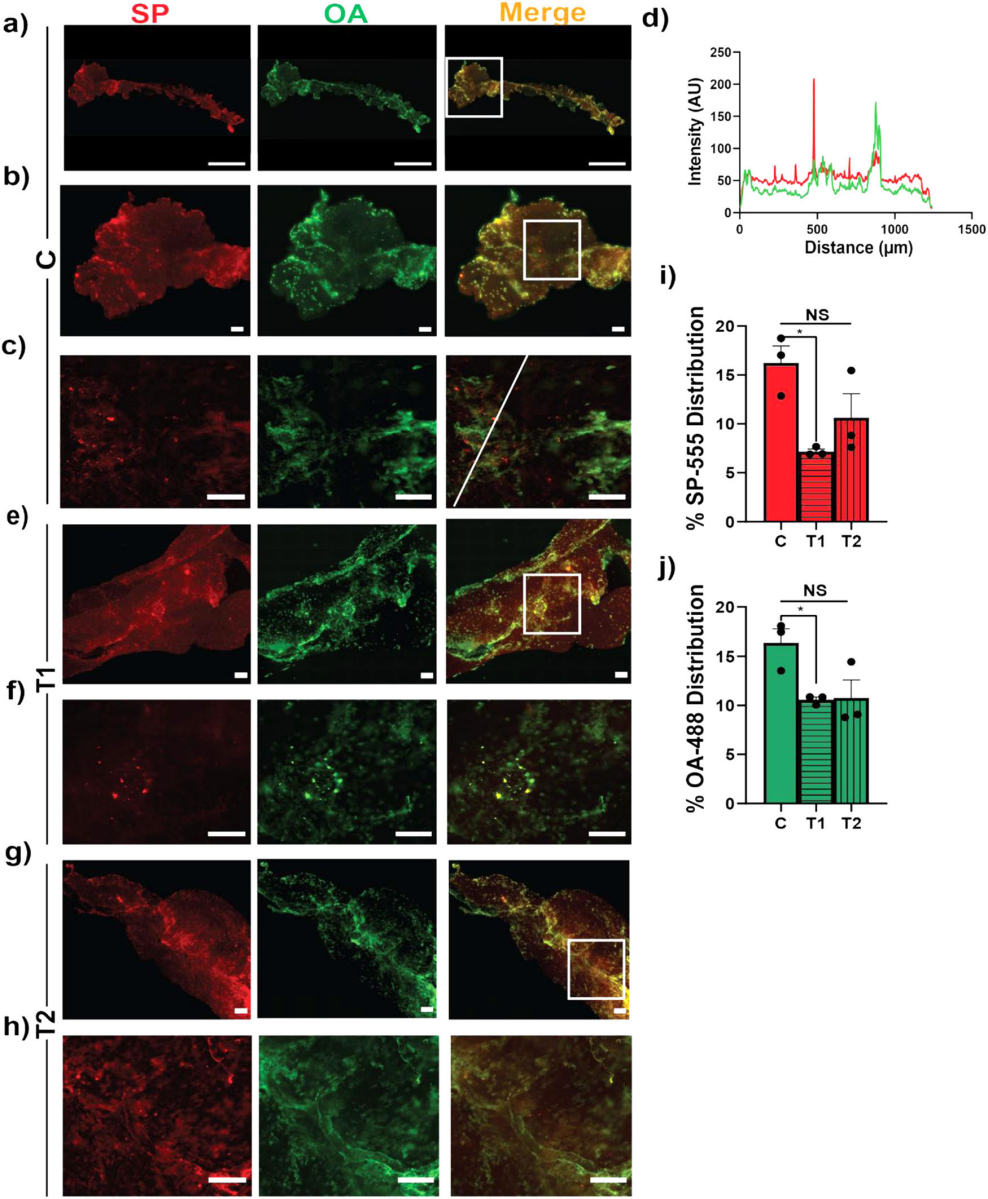
SP-555 is taken up by the isolated CP. **a**–**c** Representative images of SP-555, OA-488, and merged images for the isolated CP after incubation for 60 min. Lower panels are the magnified images of the boxed area in the upper panels. **d** Intensity plot of the line in **c**. It crosses one of the many red dots (SP-555) showing local high intensity. **e**, **f** Representative images of SP-555, OA-488 and merged images for the isolated CP after incubation with anti-ACE2 antibody. **g**, **h** Anti-SP antibody with the SP-555 and OA-488 for 60 min. Lower panels are the magnified images of the boxed areas. **i**, **j** Quantification of the SP-555 and OA-488 signal intensities for the whole areas. Scale bars: **a** (1 mm); **b**–**h** (100 μm). *N* = 3. CP was pre-incubated with T1 and T2 for 15 min and during incubation with the tracers for 60 min. Images enhanced for better presentation of details. Analysis was reformed without enhancement.

To confirm the in vivo data on SP uptake by the CP, the isolated CP was incubated with SP-555 (50 nM) and the reference molecule (OA-488), in the presence and absence of T1 or T2 for 60 min. Images confirmed that SP-555 was associated with the CP (Fig. 5a–c). SP-555 location was not always colocalized with OA-488, perhaps due to SP association with epithelial cell and resident leukocytes. Both molecules were present along vessels (Fig. 5a). In general, a similar pattern was seen for the in vivo studied, even though the CP was too folded to analyze accurately (Fig. 3e). Intensity plot across the SP-555 (red) spots revealed high intensity signal (Fig. 5d). T1 reduced the association of SP-555 with the CP, perhaps due to blocking ACE2 receptor-binding sites (Fig. 5e, f). However, T2 was less effective in reducing SP-555 association with the CP (Fig. 5g–j). This may be due to IgG/SP-555 binding to Fc receptors on leukocytes of the CP. Collectively, these data show that SP binds to the CP and indicated that T1 altered the SP-555 distribution in the CP (Fig. 5i, j).

## Discussion

Our data show that SP-NIRF, administered intravenously into young adult mice (equivalent to young mature humans), was differentially distributed to multiple organs and eliminated slowly from these regions, except the upper abdominal region (mainly liver), which showed a slow accumulation after its distribution. For the paw, SP-NIRF was shown to exponentially increased to a plateau. At 60 min post injection, regional levels were higher than that of plasma, which was undetected. Brain showed the lowest levels, whereas the lung had the highest levels followed by the kidney, heart, and liver.

Anti-SP antibody was more effective in reducing SP-NIRF multiorgan tropism than anti-ACE2, possibly due to its higher affinity for SP. These antibodies had no effects on brain SP-NIRF levels. In contrast, SP-NIRF uptake was significantly reduced with both antibodies for the lung, liver, and kidney. Although there was no significant presence of SP-555 within the brain parenchyma, blood vessels, or neuron, the CP was labeled with the injected SP-555, which may contribute to the signal detected in the isolated whole brain, but there was no detectible signal in CSF. Interestingly, both SP-555 and OA-488 was associated with the spinal cord. Thus, the brain vascular barriers (BBB and CP) restrict the entry of this viral SP into brain parenchyma, at least within 60 min.

We used the receptor-binding domain (RBD) of the SP as a surrogate of the virus (SARS-CoV-2), as RBD plays a major role in viral entry into host cells. Also, there was no significant differences in the interaction and effects of different molecular sizes of SP once they contain the RBD^32^. In addition, the transfer constant for two types of SP1 influx into brain was similar^33,34^. The RBD may have a higher affinity for ACE2 than SP of higher molecular weight^42^. We injected 57 pmol of SP, which was about 34 × 10^12^ monomeric particles using Avogadro’s number. As there are about 24 SP trimers (72 monomeric forms) on a SARS-CoV-2 virus^43^, an estimated 5 × 10^11^ viruses were injected into blood as SP. Intravenous clearance of viruses was studied at (1.6 × 10^9^ to 1.6 × 10^11^)^44^. Thus, we have used an optimum condition to study SP biodistribution, organ uptake, and elimination.

### SARS-CoV-2

SARS-CoV-2 consists of a viral lipid envelop, which encloses the nucleocapsid bound RNA^45^. The lipid membrane has structural proteins, including the SP, which forms a heterodimer (S1–S2) that is assembled as a trimer protruding from the viral surface that gives the crown-like appearance^10,45^. The S1 unit contains a RBD, which promotes attachment to the extracellular peptidase domain on ACE2 receptors on the host cell plasma membranes^10,42,43^. TMPRESS 2 (transmembrane protease, serine 2) on the host cells cleaves the SP to facilitate viral entry to cells^14,46^. There are other receptors/facilitators on the cell surface that mediate the entry of SARS-CoV-2, including sialic acid^47^ and CD147^48^.

SARS-CoV-2 enters the body mainly during inhalation. In severe COVID-19 cases, the infection is manifested as cardiopulmonary symptoms^1,4,6,49,50^. However, clinical manifestations of COVID-19 have revealed multiple organs are affected^51^, include the heart^21–23^, kidneys^24,25^, liver^26–29^, intestine^27,28,52^, and brain^3,30,31,34,35,37,53–56^. The distribution of SARS-CoV-2 RNA in autopsy tissue samples from severely infected COVID-19 patients shows evidence of multiorgan tropism^51,56^.

ACE2 is widely expressed on the cell membranes, including the gastrointestinal tract, kidneys, CP, heart, lungs, oral mucosa, and bladder^10,11,13–15,39,57^. In the brain, ACE2 mRNA is present in the cortex, striatum, hippocampus, and brain stem^16–18^. It is mainly expressed in brain regions associated with regulation of the cardiovascular system, blood pressure, and the autonomic nervous system^19^. Brain endothelium expresses ACE2 as the protein^32^ and as the RNA-sequencing^11,58^, and the SARS-CoV-2 protease cathepsin B^40^. ACE2 is expressed on pericytes^12^. The wide expression of ACE2 receptor suggests that many organs will be affected by SARS-CoV-2.

### SP biodistribution from the blood to brain

The mammalian CNS is unique in that it is enclosed within its vascular barriers and has CSF continuously circulating around it^59–62^. The vascular barriers at the blood–brain interface (the BBB) and blood–CSF interface (blood–CSF barrier; the CPs) restrict the diffusion of polar molecules and large molecules into and out of the brain^59–62^. The physical sites of the BBB and blood–CSF barrier are the tight junctions between endothelial cells and epithelial cells, respectively^63,64^, which restrict paracellular diffusion. The endothelium of the cerebrovasculature is at the interface between the blood and brain^59,60^, and SP needs to interact with the luminal surface of the endothelium to enter brain from blood.

CSF is mainly produced within the cerebral ventricles by the CPs and circulate from the lateral ventricles to the subarachnoid space, around the brain, spinal cord, and within the spinal canal, before ultimately draining at multiple outflow sites into blood^61,62,65–68^. A major CSF drainage pathway is via the olfactory bulb, across the cribriform plate and towards the cervical lymphatic nodes, and the spinal cord^38,61,62^. The endothelium of the CP is fenestrated, and allows entry of proteins into the stroma, which is restricted from entering CSF due to the tight junctions between the epithelial cells at the apical surfaces^59,61,62^.

Following intravenous SP-NIRF injection, it will be distributed to all organs via blood, and uptake determined by the degree of restriction offered by the endothelium of the vasculature^69,70^. Continuous endothelium of the CNS cerebrovasculature offers the greatest restriction to protein flux, except at the CP^59–62^. Thus, both SP-555 and OA-488 will cross the fenestrated endothelium of the CP, but not the epithelium layer, which has tight junctions between these cells at the apical side^61^, as seen in our data. Although the CP was labeled with SP-555, there was no detection of SP-555 in CSF, as the epithelium, likely, restricts its entry.

However, there was SP distribution to the spinal cord, which may reflect the greater permeability of the blood-spinal barrier to large (albumin) and small (inulin) molecules compared to brain^41^. Thus, the brain vascular barriers at the BBB and CP seem to restrict the entry of SP into brain and CSF from blood. There is a report that SP interacts with the brain endothelial cells using in vitro models of the BBB^32^. In addition, there is a report that SP enters the murine brain from blood^33,34^. Similar to this report, we found that the SP-NIRF brain levels were not dependent on ACE2. The time point for these reports and our study was similar. In our study, the low levels of SP-NIRF in brain is likely associated with the CP. Thus, our data support the finding that CP is a potential target of SARS-COV-2. SARS-CoV-2 was shown to be associated with the CP, which can damage the epithelium^71^, and SARS-CoV-2 transcripts were associated with the CP^54^. We found that SP-555 also binds to the apical surface of the CP using the isolated CP from mice, which may also limit SARS-CoV-2 in CSF. The highly convoluted apical surface of the CP and its folding make it difficult to analyze tracer distribution. However, there are spots of high intensity SP-555 not associated with OA-488. In addition, there are reports both for the presence and absence of SARS-CoV-2 in the CSF^5,53,72,73^. Further work is needed.

The exact mechanism of SARS-CoV-2 neuroinvasion is unclear. Viruses could enter the brain by retrograde transport via sensory nerve endings within the nasal and buccal cavities^74–77^, and possibility via the gastrointestinal tract^53,78^. It is possible that SARS-CoV-2 enters the brain as a consequence of pneumonia-induced hypoxia. However, there are reports of encephalitis, which was not due to COVID-19-induced hypoxia^3,79,80^. The virus was detected in frontal lobe brain tissue, which may suggest access via the nasal route^81^. There are also reports of the presence of SARS-CoV-2 in autopsy brain samples and especially in the olfactory mucosal region of severely infected patients^75–77,82^. However, this is not always the case, as SARS-CoV-2 RNA was not detected in the nasopharyngeal swab of a patient but was detected in CSF^5^. In a COVID-19 patient with meningitis the virus RNA was detected in the CSF^3^. Viral particles are associated with the cerebrovasculature and the endothelium of other organs^83–87^. However, it is unclear whether these clinical neurological presentations seen in severe COVID-19 patients are due to the virus entering brain or as a consequence of cardio-respiratory, multiorgan failure, and systemic inflammation. It is possible that the viral invasion of the lungs and the subsequent inflammation will dominate and drive the outcomes of the infection. Thus, further studies are needed to support significant neuroinvasion as the explanation for the neurological symptoms associated with COVID-19.

### SP biodistribution from the blood to peripheral organs

We performed in vivo dynamic imaging in regions (ROIs) that were known to be affected by COVID-19. Although these ROIs are representing multiple organs/tissues, in some of them signals were associated with a predominant organ. These included the neck region where the SP-NIRF signal was predominantly from the salivary gland and the upper abdomen where it was from the liver. For the thoracic ROI, the NIRF signal was most likely from the lungs and heart, whereas the signals from the lower abdomen are from mainly the intestine and kidneys. Signals from the paw are due to the hairless skin.

There is a continuous endothelium with tight junction between endothelial cells for the lung which should limit protein flux^88–90^. However, unlike the brain, the lungs had the highest SP-NIRF uptake from blood. This may be due to the heterogeneity of the endothelial cells and SP-NIRF binding to the glycocalyx and possibly ACE2 receptor^70,88–90^. For the liver, the sinusoidal endothelium and Kupffer cells clear viruses and proteins^44,91–94^. ACE2 is expressed on many parts of the nephron, including the luminal brush border of the proximal tubule^95–98^. Thus, the kidney likely takes up a significant level of SP. Also, ACE2 is expressed in the heart^21–23,97,99^. Our findings that SP is distributed to the salivary glands may support suggestions that these glands are a potential reservoir of SAR-CoV-2, which may lead to parotitis-like syndrome^100,101^. Interestingly, within the gastrointestinal tract, blood-borne SP-NIRF was mostly located at the duodenum^27,28,52,102,103^. The intestine expresses ACE2 and this has been suggested to contribute to COVID-19-related gastrointestinal tract effects^27,28,52,102^. Our data confirm multiorgan tropism seen in clinical manifestations of COVID-19, with the exception of brain in young healthy mice.

### Role of anti-ACE2 and anti-SP antibodies in SP biodistribution

Although the antibodies were injected 15 min prior to the tracer injection, they will remain in the circulation for a longer time as the half-life is very long, about 18–20 hrs^104^. In mice, many organs are involved in IgG clearance, including the liver, kidney, muscle, skin, and spleen^104,105^. The rationale for the current study is that for the anti-ACE2 antibody, it will likely interact with ACE2 receptor and reduce SP binding to the host cells^106^. In contrast, anti-SP antibody will interact with the SP and reduce the free SP levels, which in turn will reduce SP binding to tissues. Our data show that the anti-SP antibody was effective in reducing SP biodistribution and uptake into peripheral organs^107^. In contrast, there was no effect of these antibodies on brain SP-NIRF uptake. In addition, although mouse ACE2 may not bind SP as efficiently as humanized ACE2^108–110^, at the higher SP concentrations it seems to interact with mouse ACE2. This is a likely explanation for anti-ACE2 antibody less effectiveness in mice. It is possible that IgG-SP could bind to Fc receptors on cells, especially leukocytes. The CP has resident immune cells, including macrophages and CD4 cells^111,112^, which could have a greater effect on SP-NIRF uptake by the isolated CP, which may not be seen in vivo due to clearance by the circulation and immune cells.

### Limitations of the present study

We used the RBD of the SP as a surrogate of the virus (SARS-CoV-2) to study its biodistribution and elimination, as RBD plays a major role in the viral entry into host cells. However, the distribution pattern might be different for the actual virus and the molecular weight of SP would influence filtration at the kidneys and convective flow into peripheral organs, such as muscles. Thus, a lower molecular weight RBD (<albumin) likely represent a maximum biodistribution. The biodistribution of SP was not systematically evaluated in all organs/cells, such blood cells, pancreas, and others. Therefore, it is possible that an organ not studied may also play a role in SP uptake. However, these organs may not be as significant, since there was no significant SP-NIRF level in the rest of the mouse. Thus, we focused on organs with significant SP signal. Nevertheless, our studies and that of other, using the SP, could provide critical data to better and safely model the virus behavior in a host before using the virus. In the current study organ biodistribution and the efficacy of anti-SP antibody were assessed and thus, intravenous injection of SP was used. However, the main route of viral entry into the body in inhalation. In terrestrial animals, injection of fluid into the nose is not physiological. The SP would need to be formulated into an aerosol for inhalation studies, which is beyond the scope of this study. In addition, high levels would be needed so that enough can enter blood via inhalation to meet the objectives of the current study. In patients, blood levels of SARS-CoV-2 are low^65^. Plasma profile of SP was not possible as serial sampling of large volumes of plasma would be needed. The total blood volume is ~1.5 ml (in a 20 g mouse and assuming blood volume is 78 ml/kg body weight (vivarium)) and plasma volume ~0.9 ml. However, the same dose was administered intravenously and all mice were of the same age and sex.

## Conclusions

SP-NIRF is differentially distributed to many organs, which may explain SARS-CoV-2 multiorgan tropism, and this may contribute to organ failure, in addition to respiratory failure, in young adults. The lungs had the highest SP levels and the brain the lowest. There was significant SP signal in the peripheral organs, including the salivary glands and intestine, and the spinal cord and CP, but not in CSF. Thus, the brains vascular barriers were effective in restricting the flux of SP (protein) from blood into the brain parenchyma. The differential SP organ uptake is likely determined by, mainly, ACE2 levels. Anti-SP antibody was more effective than anti-ACE2 antibody in suppressing SP biodistribution and organ uptake, possibly due to a greater affinity for the anti-SP antibody for SP and lower affinity for the mouse ACE2. Thus, therapies, include passive immunity, using anti-SARS-CoV-2 antibodies and convalescent plasma, which likely contains anti-SARS-CoV-2 antibodies, should be effective in reducing SARS-CoV-2 biodistribution, and thus COVID-19 severity. It also offers confirmation that vaccines against the SP, which generates anti-SP antibodies, are likely to neutralize the virus tissue distribution and minimize severity of this infection. Further work is needed in older mice and with systemic inflammation.

## Methods

### Materials

SARS-CoV-2 SPs (recombinant SARS-CoV-2 SP (S-RBD; cat# RP-87678, HEK293 cell expressed and binds ACE2) and ovalbumin Alexa Fluor-488 conjugate (Cat# 034781; molecular weight 45 kDa) were obtained from Life Technologies Corporation, Carlsbad CA, USA. The SP (molecular weight 36 kDa) was labeled with NIRF using a kit (IRDye800CW Microscale kit, Li-COR Biosciences, Nebraska, USA) and by following the manufacturer’s instructions. This conjugation forms a stable amide bond and has been used in clinical trials (Li-COR Biosciences). SP was labeled separately with Alexa Fluor 555 using a kit (Microscale protein labeling kit; ThermoFisher Scientific; Waltham, MA, USA) and by following the manufacturer’s instructions. In addition, both labels were purified using 3 kDa molecular weight cutoff ultrafiltration filter (Amicon Ultra Centrifugal Filter, Millipore). There was no detectable dye in the filtrate. Anti-SARS-CoV-2 SP antibody (SARS-CoV-2 Spike Protein Monoclonal Antibody (cat# MA5-36087; immunogen SARS-CoV-2 SP that interact with HeLa cell expressed SARS-CoV-2) and anti-ACE2 antibody (ACE2 Recombinant Rabbit Monoclonal Antibody (Cat# MA532307; interact with mouse ACE2) were obtained from Life Technologies Corporation.

### Mice

C57BL/6J (2–3 months old; Jackson Laboratory; Bar Harbor, ME, USA), male mice were used. All animal studies were performed in accordance with the National Institute of Health guidelines and using protocols approved by the University Committee on Animal Resources. Mice were housed in the vivarium of the University of Rochester, School of Medicine and Dentistry, on a 12 : 12 light/dark schedule (6:00 a.m.–6:00 p.m.) with food and water ad libitum.

### ACE2-SP-555 binding

ACE2 (recombinant mouse (HEK293cells; cat#230-30172) and recombinant human ACE2 (HEC 293 cells; cat# 230-30165), RayBiotechLife, Inc., Peachtree Corners, GA), dissolved in carbonate/bicarbonate buffer, was immobilized (25 μg ml^−1^) on glass slides for 1 h at room temperature (RT), blocked with a non-protein buffer (Pierce Protein-free (PBS) blocking buffer, Cat# 37572, ThermoFisher Scientific), washed, incubated with SP-555 at different concentrations (0.01–1 μg ml^−1^) in Hank’s buffered salt solution (HBSS) for 1 h at RT, washed, mounted, and imaged. SP-555 intensity from ten fields in two slides for each concentration were analyzed and expressed as intensity per μm^2^.

### Intravenous injections

Mice were anesthetized with isoflurane, as it has fewer systemic hemodynamic effects^113^, and were placed on a temperature-controlled stage to maintain body temperature. Anesthesia was maintained with 1–2% isoflurane in oxygen. Once anesthetized, a midline incision from the neck to the pelvic area was made and the skin retraced to enhance the NIRF signal intensity. Local anesthetic (Topical Lidocaine 4% gel, ESBA Laboratory, Inc., FL, USA) was applied to the exposed regions, which was moistened with saline. This also exposed the right external jugular vein for a minimal invasive intravenous injection using a 30 G needle connected to a 500 µL insulin syringe via a tubing. The tubing contained the fluorescent tracer (10 μL), which was the SP conjugated to IRDye800CW (SP-NIRF; 5.7 pmol μL^−1^ (2.05 μg ml^−1^)), or SP conjugated to Alexa-555 (SP-555; 5.7 pmol μL^−1^) and a protein reference molecule of similar molecular weight (ovalbumin-488 (OA-488, 0.1%), dissolved in PBS (pH 7.4). The injection contained 10 μL PBS, 1 μL air gap, 10 μL tracer, 1 μL air gap, and 50 μL PBS to wash in the tracers completely. The injection was completed in 1 min. We used NIRF to minimize the natural background fluorescence of biomolecules, increase the signal to noise signals, and to provide a better contrast between the target and background^38^. We also used SP-555 to visualize and map its biodistribution in tissue sections and compared this to that of a reference protein molecule of similar molecular weight (OA-488).

The groups of mice were as follows: young male mice (2–3 months old), T1 (anti-ACE2 antibody; young male mice (2–3 months old)), and T2 (anti-SP antibody; young male mice (2–3 months old)). T1 (10 μg) or T2 (10 μg) was injected instead of the 10 μL PBS 15 min prior to the tracer (the same dose). The experimenter was blinded to the experiment design and unaware of the tracers T1 and T2 used in these studies.

### In vivo dynamic imaging

NIRF intensities were measured using a custom-made NIR system (Supplementary Fig. 1a). Mice were placed on a heated surface (Indus Instruments, Webster, TX, USA) and the hair was removed with a depilatory cream before surgery. The imaging system was composed of a lens (Zoom 7000, Navitar, Rochester, NY, USA), a NIRF filter set (Semrock ICG-B, IDEX Health & Science LLC, Rochester, NY) and camera (Prosilica GT1380, Allied Vision Technologies, Exton, PA, USA). NIRF was excited with a tungsten halogen bulb (IT 9596ER, Illumination Technologies, Inc., Syracuse, NY, USA) through a ring illuminator (Schott, Elmsford, NY, USA). Imaging settings and recordings were accomplished through a custom-built LabVIEW program (National Instruments, Austin, TX, USA). Real-time NIR imaging was performed before injections (background) and every 2 s for 60 min after SP-NIRF injection into the right jugular vein to quantify its biodistribution^38,114^. Using ImageJ software (National Institutes of Health, Bethesda, MD, USA), ROIs were identified and the ROI fluorescence intensity recorded at the same settings, as reported^38^. The person analyzing the data were blinded to the experimental design.

### Tissue preparation for fluorescence imaging

After the duration of the experiment, mice were transcardially perfused using cold PBS and paraformaldehyde (PFA) (4% in PBS pH 7.3). Tissues samples were removed, stored overnight in PFA at 4 °C, and re-stored in cold PBS. Brain was cut into 100 µm coronal sections using a vibratome (Leica VT1000E). Spinal columns (the cervical region), liver, kidney, lungs, and heart were embedded in Optimal Cutting Temperature compound and cut into 30 μm sections using a cryostat. In some experiments, the skin was removed from the head, then decalcified in 13% EDTA for 5 days, and cut using a cryostat. Sections were mounted on Superfrost Plus glass slides using ProLong Gold Antifade Mountant medium (ThermoFisher Scientific, Waltham, MA, USA) for fluorescence imaging (VS120 Virtual Slide Microscope, Olympus). Exposure and gain were fixed for all experimental groups based on pilot experiments. All fluorescence (SP-555 and OA-488) quantification was performed without enhancement of signals. The person performing the imaging and analysis were blinded to the experimental design and tracer used. This was finally decoded when the figures were prepared for publication.

### Immunohistochemistry

Brain sections were stained for neurons (NeuN) and blood vessels (collagen IV), and kidney sections for ACE2. The tissue was blocked with 5% donkey serum for 1 h at RT and the primary antibodies incubated overnight. The primary antibodies were mouse anti-NeuN (1 : 200, Cat# MAB377, Millipore Sigma, Billerica, MA, USA), anti-collagen IV (1 : 200, Cat# SAB4500369, Millipore), and anti-ACE2 antibody (1 : 100; Life Technologies. Cat# MA532307). The secondary antibodies were donkey anti-rabbit-Cy5 for collagen IV and ACE2, and donkey anti-mouse-Cy5 for the NeuN (Jackson ImmunoResearch, Inc., West Grove, PA, 1 : 500), which were added and incubated for 1.5 h at RT. Immunofluorescence was visualized by using an epifluorescence microscope (VS120 Olympus).

### Ex vivo NRIF imaging of whole tissues

At the end of in vivo imaging, animals were killed and tissue samples collected, including the brain, spinal column, liver, lungs, kidneys, heart, spleen, and intestine. Tissues were washed equally and imaged individually on the ventral and dorsal surfaces using the same parameters as for in vivo imaging. The average tracer intensities were used as there was no significant difference in the dorsal and ventral values).

### Ex vivo fluorescence imaging of tissue sections

Ex vivo epifluorescence microscopy was used to evaluate the degree of tracer distribution in the tissue sections. Exposure and gain were fixed for all experimental groups based on the pilot experiments. To quantify the extent of tracer intensity distribution in whole tissue sections, the whole-slice/section images were analyzed using ImageJ (National Institutes of Health, imagej.nih.gov/ij/), and Qupath (https://qupath.github.io/). In Qupath, ROI was created for each analyzed sample. A training image was created from the ROIs to train a three-way random trees pixel classifier to account for variation and distinguish between positive fluorescence and negative background. Separate pixel classifiers were created for 555 and 488 nm channels. For each slice, fluorescence emission channels were split into individual channels of 8-bit TIFF grayscale images and a whole-section ROI was defined. The pixel area of positive fluorescence (threshold pixel intensity for both green (488 nm) and red (555 nm) channel was calculated for each section, and the average expressed as a percentage of the whole-section area. The Olympus VS120 Virtual Slide Microscopy was used to identify colocalization of immunolabeled (cellular or vascular components) with the injected tracer.

### Kinetics analysis

For each experiment, image sequences were imported into ImageJ. The ROIs, identified from pilot experiments, were neck (including the salivary glands, trachea, pharynx), thorax (lungs and heart), upper abdomen (including the liver, spleen), lower abdomen (the intestine and kidneys but not bladder), and paw (a type of skin region with no clear relationship with COVID-19) (Fig. 1a). Mean pixel intensity within each ROI was measured for each time point using the images without enhancement. The background signal was the same ROI without the injection and this was subtracted from the intensity at each time point. To correct for experimental variation, the intensity at each time point was divided by peak intensity to standardize the data. Peak intensity (Imax) was identified as the average of 10 s at the peak identified using GraphPad Prism software (Version 9.1.0 (216)). The AUC was determined by running AUC analysis option in GraphPad Prism. The elimination rate constant was determined from the slope of the falling phase of the intensity profile using a semi-natural logarithmic (Ln) plot and the linear regression function in the GraphPad Prism. For the liver, since there was a linear component of the intensity-time profile after the distribution phase, the slopes were determined from the plots using the linear regression option in GraphPad Prism.

### CSF and blood samples

In a separate group of experiments, after tracer injection (young mice), CSF and blood samples were collected. CSF was collected from the cannulated cisterna magna (to minimize contamination from blood) and samples from two mice pooled to get a volume to analyze (15 μL). Blood samples was collected by cardiac puncture (100 μL). A fixed volume (15 μL) of plasma and CSF in an Eppendorf tube was used to determine NIRF intensities and for comparison. Background was the same volume of saline an Eppendorf tube. The total volume of plasma in a mouse is <1 ml.

### CP uptake

In a separate group of experiments, young mice were perfused with cold PBS and the CPs isolated from the lateral ventricles. These were incubated in 100 μL of oxygenated (100% O_2_) HBSS and in a humidified oxygenated chamber at 25 °C for 60 min. There were three groups: control, T1, and T2. The traces were 50 nM SP-555 and the reference molecule OA-488 (0.01%). T1 or T2 (100 nM) were added 15 min before the tracers. At the end of the experiment, the incubation media were removed, CP washed 3 times with 500 μL cold PBS, fixed in 100 μL 4% PFA, washed three times with 500 μL cold PBS, mounted on glass slides, and imaged.

### Statistical analysis

Data were analyzed by analysis of variance followed by post hoc Tukey’s test using GraphPad Prism. Differences were considered to be significant at *p* < 0.05. For statistical representation, *P < 0.05, **P < 0.01, ***P < 0.001, and ****P < 0.0001 are the levels of statistical significance. NS is not significant. All values were expressed as mean ± SEM. *N* = 4 mice (Power analysis). Student’s *t*-test with Welch’s correction was used for sample comparison. GraphPad Prism software (version 9.1.0 (216)) was used for all analysis.

### Reporting summary

Further information on research design is available in the Nature Research Reporting Summary linked to this article.

## Supplementary information

Supplementary Information

Reporting Summary

## Data Availability

The data set is available from the corresponding author on reasonable request. The source data for the figures are also available. Also, these can be found at https://figshare.com/s/57f33a02cb98f9f0e2ec
