# Supplementary material for: Spike protein multiorgan tropism suppressed by antibodies targeting SARS-CoV-2
Source: Commun Biol. 2021 Nov 22;4:1318. doi: 10.1038/s42003-021-02856-x (PMC8609008; doi:10.1038/s42003-021-02856-x)
Supplement: Supplementary file 1 — Supplementary Information [file 42003_2021_2856_MOESM1_ESM.pdf]

Spike protein multiorgan tropism suppressed by antibodies targeting SARS-CoV-2

Authors: Molly Brady[1], Conor McQuaid[1], Alexander Solorzano[1], Angelique Johnson[1], Abigail Combs[1], Chethana Venkatraman[1], Akib Rahman[1], Hannah Leyva[1], Wing-Chi Edmund Kwok[2], Ronald W Wood[1,3], Rashid Deane[1, *]

Del Monte Institute of Neuroscience Department of Neuroscience[1], Departments of Imaging Sciences[2], Obstetrics and Gynecology[3], Urology[3], University of Rochester, URMC, 601 Elmwood Avenue, Rochester, NY 14642, USA

*Corresponding author: Rashid_Deane@urmc.rochester.edu

Supplementary Figure 1

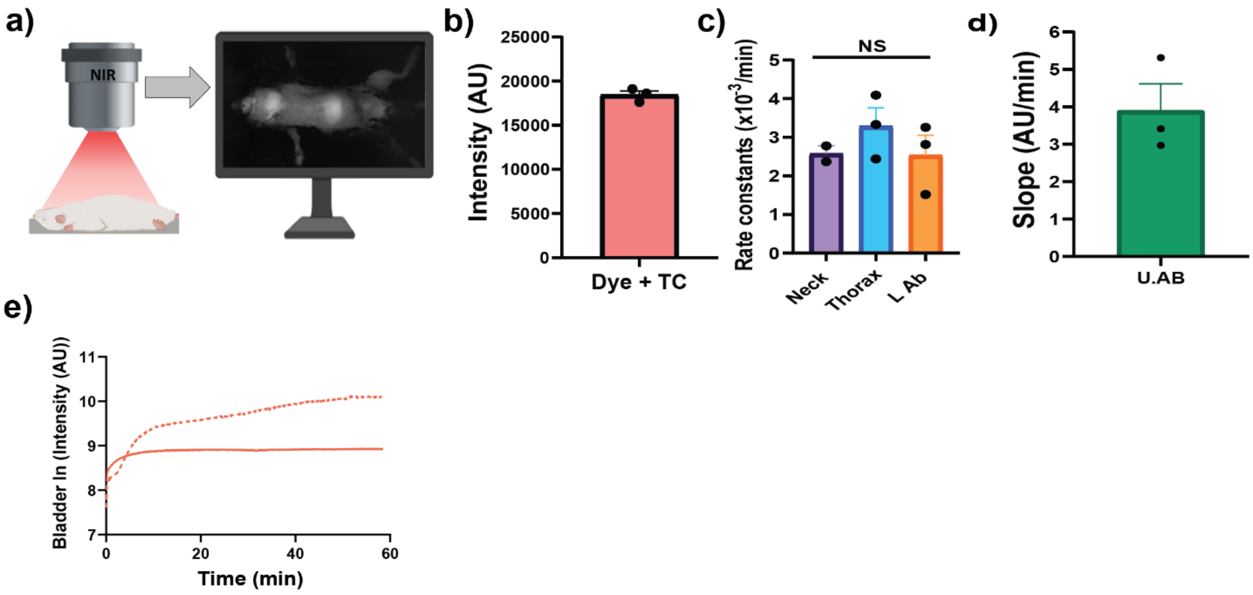

Supplementary Figure. 1. External SP-NIRF in vivo dynamic imaging

a) SP-NIRF(SP-near infrared fluorescence) imaging system for the whole mice. **b**) Confirmation that NIRF signal can be detected from under the isolated mouse rib cage (TC). 10 μL NIRF sample was placed in an Eppendorf tube and covered with the isolated rib cage from a non-injected mouse. **c**) Elimination rate constants for SP-NIRF calculated for ROIs with disappearance part of the profile using the semi-Ln intensity-time profiles. **d**) Slope for the rising phase after SP-NIRF distribution for the upper abdomen (U.Ab). **e**) Representative data showing variations in urinary

bladder SP-NIRF levels in control mice. Values are mean ± SEM, N=5 young male mice. AU (arbitrary units).

Supplementary Figure 2

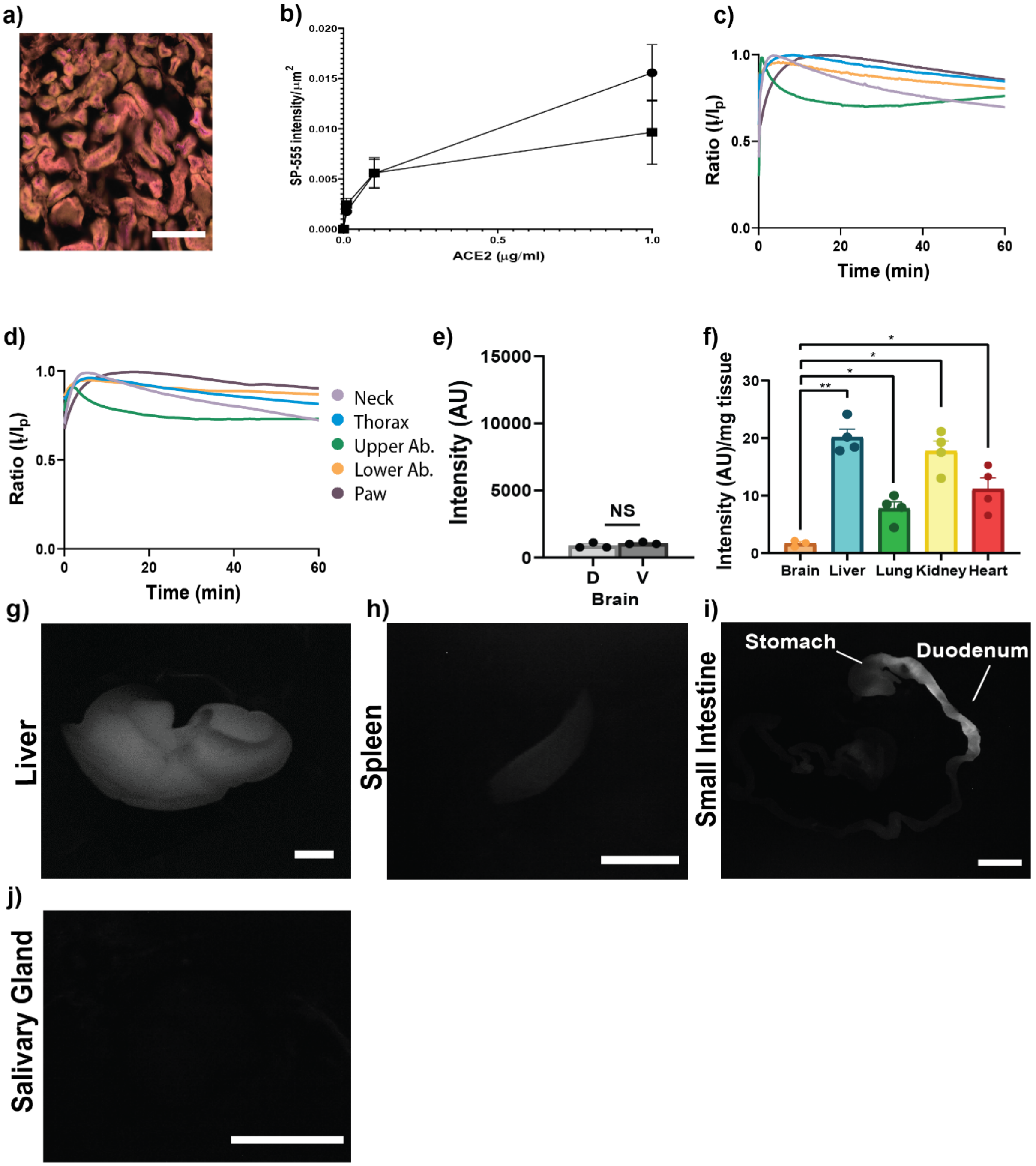

Supplementary Figure. 2. SP-NIRF biodistribution and elimination

a) Representative image confirming anti-ACE2 antibody-ACE2 interaction (purple) in mice kidneys. **b**) SP-555 binds to both mouse (square) and human (circle) ACE2. N= 10 fields from 2 slides for each data point. **b-c**) Standardization of data by dividing intensities at each time point by the peak intensity (I_T/I_P ratio) for anti-ACE2 antibody,T1 (**b**) and anti-SP antibody,T2 (**c**). **d**) SP-NIRF intensity for the dorsal (D) and ventral (V) brain surfaces. **e**) SP-NIRF intensities.mg^{-1} wet weight for organs at 60 minutes. **f-i**) SP-NIRF images for the liver (**f**), spleen (**g**), intestine (**h**) and salivary gland (**i**). Values are mean ± SEM, N=4 young male mice. AU (arbitrary units). Scale bar a (0.3 mm); f-i (1 mm).

Supplementary Figure 3

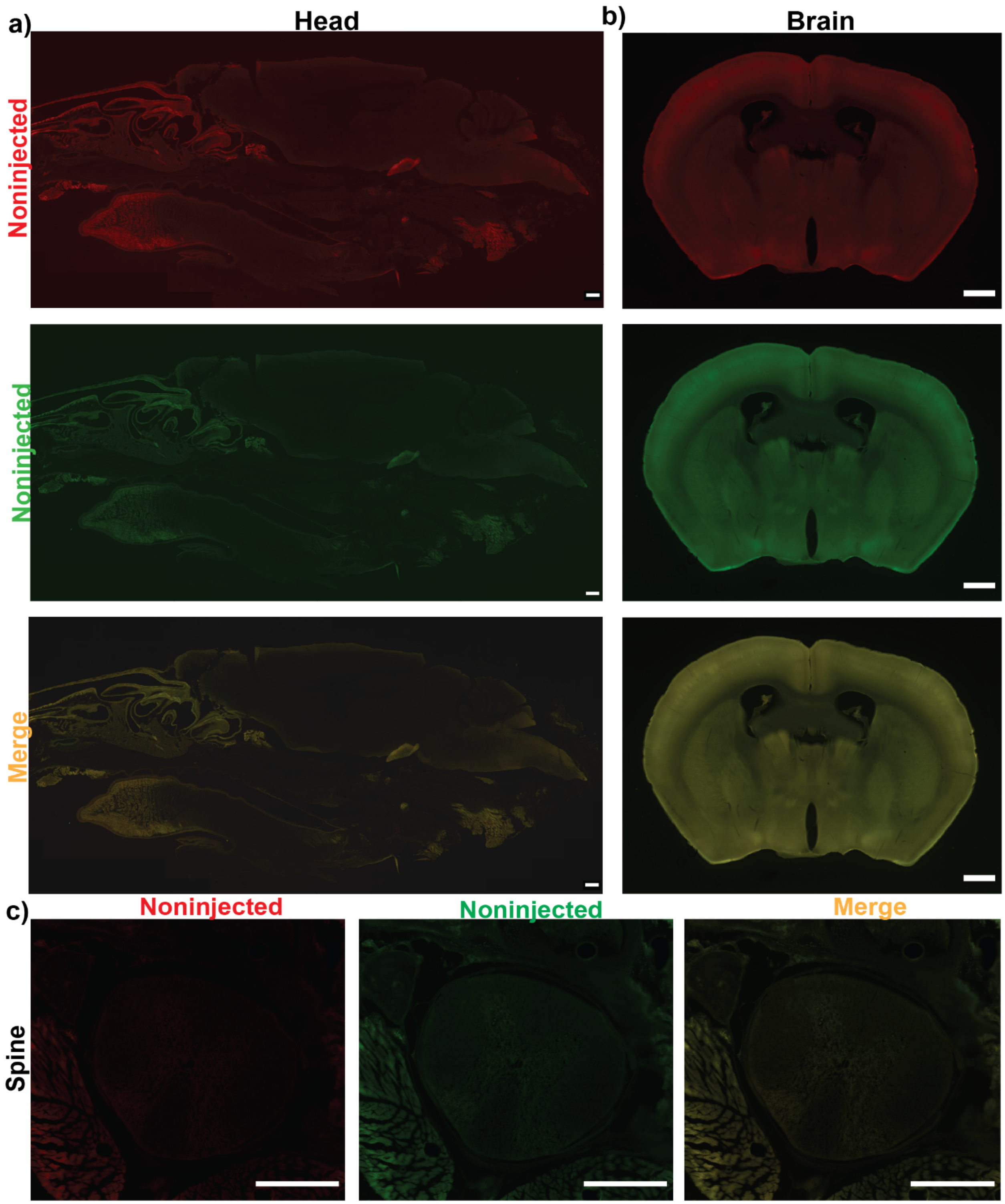

Supplementary Figure. 3. CNS autofluorescence

a-b) Representative sagittal section of the head (**a**) and coronal brain section (**b**) for a young mouse showing autofluorescence at wavelengths 555 and 488 nm and merged images. **c**) Representative image of the spine with spinal cord. N=3. Scale bar: a-b (1 mm); c (0.5 mm). Images not enhanced.

Supplementary Figure 4

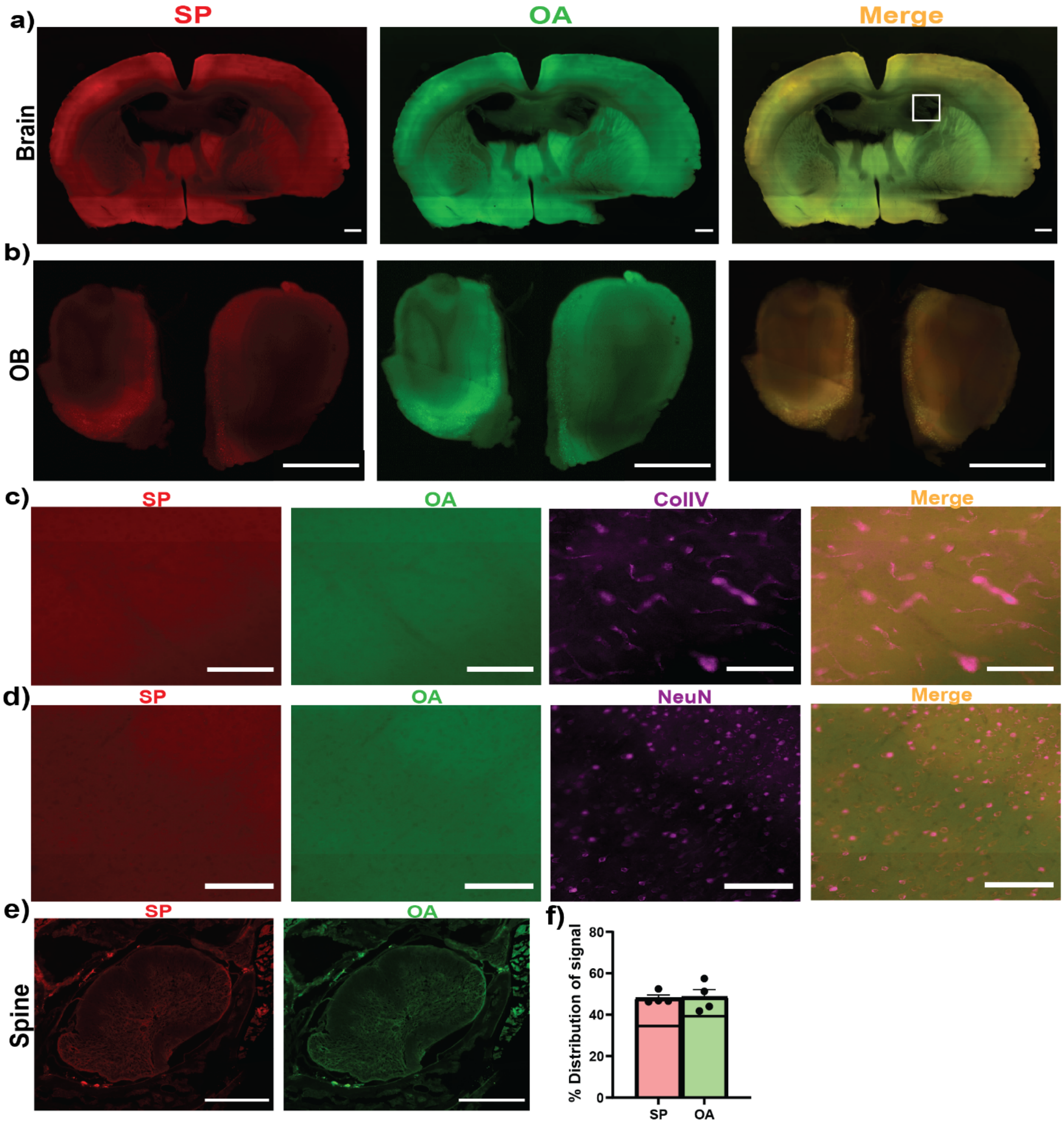

Supplementary Figure. 4. SP-555 is not present in brain parenchyma.

a-b) Representative coronal sections of brain (**a**) and olfactory bulb (**b**) showing no significant levels of SP-555 or OA-488 compared to autofluorescence. **c-d**) SP-555 in not present in collagen IV-positive vessels (**c**) or NeuN-positive neurons (**d**). **e**) Spinal cord shows some present of the tracers. **f**) Quantification of SP-555 and OA-488 distribution area for the spinal cord only. Lines on the columns are the levels of auto fluorescence of the spinal cord from non-injected mice. N=3-4. Scale bar: a-b (1 mm); c-d (100 μm); e (0.5 mm). Images enhanced for presentation of the details.

Supplementary Figure 5

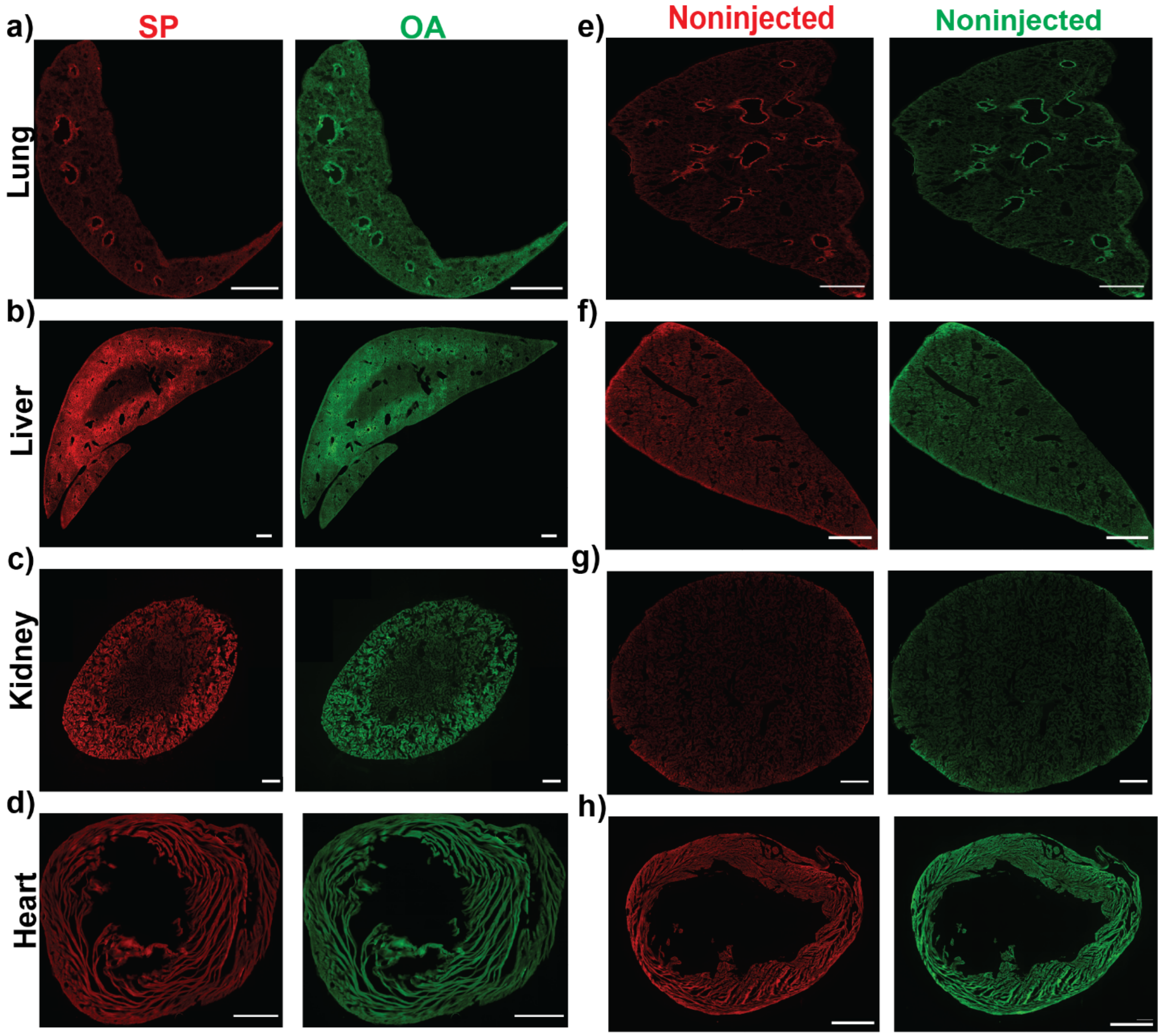

Supplementary Figure. 5. SP-55 and OA-488 are present in peripheral organs.

A-D) Representative sections (SP and OA) of lung (**A**), liver (**B**), kidney (**C**) and heart (**D**) showing higher levels of SP-555 or OA-488, except for the heart, compared to autofluorescence. **E-H**) Representative images of the lung (**E**), liver (**F**), kidney (**G**) and heart (**H**) showing autofluorescence at wavelengths 555 and 488 nm in non-injected mice. N=3-5. Scale bar: A-H (1 mm).Images not enhanced
