# Supplementary material for: Spike protein multiorgan tropism suppressed by antibodies targeting SARS-CoV-2
Source: Commun Biol. 2021 Nov 22;4:1318. doi: 10.1038/s42003-021-02856-x (PMC8609008; doi:10.1038/s42003-021-02856-x)
Supplement: Supplementary file 2 — Reporting Summary [file 42003_2021_2856_MOESM2_ESM.pdf]

nature portfolio

Corresponding author(s): R. Deane

Last updated by author(s): Oct 21, 2021

Reporting Summary

Nature Portfolio wishes to improve the reproducibility of the work that we publish. This form provides structure for consistency and transparency in reporting. For further information on Nature Portfolio policies, see our Editorial Policies and the Editorial Policy Checklist.

Statistics

For all statistical analyses, confirm that the following items are present in the figure legend, table legend, main text, or Methods section.

n/a	Confirmed	
☐	☒	The exact sample size (n) for each experimental group/condition, given as a discrete number and unit of measurement
☐	☒	A statement on whether measurements were taken from distinct samples or whether the same sample was measured repeatedly
☐	☒	The statistical test(s) used AND whether they are one- or two-sided<br>*Only common tests should be described solely by name; describe more complex techniques in the Methods section.*
☒	☐	A description of all covariates tested
☒	☐	A description of any assumptions or corrections, such as tests of normality and adjustment for multiple comparisons
☐	☒	A full description of the statistical parameters including central tendency (e.g. means) or other basic estimates (e.g. regression coefficient) AND variation (e.g. standard deviation) or associated estimates of uncertainty (e.g. confidence intervals)
☒	☐	For null hypothesis testing, the test statistic (e.g. F, t, r) with confidence intervals, effect sizes, degrees of freedom and P value noted<br>*Give P values as exact values whenever suitable.*
☒	☐	For Bayesian analysis, information on the choice of priors and Markov chain Monte Carlo settings
☒	☐	For hierarchical and complex designs, identification of the appropriate level for tests and full reporting of outcomes
☒	☐	Estimates of effect sizes (e.g. Cohen's d, Pearson's r), indicating how they were calculated

Our web collection on statistics for biologists contains articles on many of the points above.

Software and code

Policy information about availability of computer code

Data collection	N/A
Data analysis	N/A

For manuscripts utilizing custom algorithms or software that are central to the research but not yet described in published literature, software must be made available to editors and reviewers. We strongly encourage code deposition in a community repository (e.g. GitHub). See the Nature Portfolio guidelines for submitting code & software for further information.

Data

Policy information about availability of data

All manuscripts must include a data availability statement. This statement should provide the following information, where applicable:

- Accession codes, unique identifiers, or web links for publicly available datasets
- A description of any restrictions on data availability
- For clinical datasets or third party data, please ensure that the statement adheres to our policy

Data availability statement: There are three options. All data analysed during this study are included in this published article and its supplementary information files. Also, the dataset is available from the corresponding author on reasonable request. In addition, the datasets for the current study are available in the Figshare depository (DOI: 10.6084/m9.figshare.16838110 after publication), and at present there is a link (https://figshare.com/s/57f33a02cb98f9f0e2ec).

Field-specific reporting

Please select the one below that is the best fit for your research. If you are not sure, read the appropriate sections before making your selection.

☒ Life sciences ☐ Behavioural & social sciences ☐ Ecological, evolutionary & environmental sciences

For a reference copy of the document with all sections, see nature.com/documents/nr-reporting-summary-flat.pdf

Life sciences study design

All studies must disclose on these points even when the disclosure is negative.

Sample size	Sample size was determined using power analysis from pilot experiments, and from experience using mice to generate data, with a view to use the minimum animal for research
Data exclusions	There were no Dat exclusion for this study
Replication	Repeat the same experiment mice
Randomization	For each group the mice were randomly place into three groups
Blinding	The experimenter, and the person do the imaging and analysis was blinded to the tracer used and to the treatments (T1 and T2)

Reporting for specific materials, systems and methods

We require information from authors about some types of materials, experimental systems and methods used in many studies. Here, indicate whether each material, system or method listed is relevant to your study. If you are not sure if a list item applies to your research, read the appropriate section before selecting a response.

Materials & experimental systems

n/a	Involved in the study	
☐	☒	Antibodies
☒	☐	Eukaryotic cell lines
☒	☐	Palaeontology and archaeology
☐	☒	Animals and other organisms
☒	☐	Human research participants
☒	☐	Clinical data
☒	☐	Dual use research of concern

Methods

n/a	Involved in the study	
☒	☐	ChIP-seq
☒	☐	Flow cytometry
☒	☐	MRI-based neuroimaging

Antibodies

Antibodies used	Anti-SARS-CoV-2 spike protein antibody (SARS-CoV-2 Spike Protein Monoclonal Antibody (cat# MA5-36087; immunogen SARS-CoV-2 spike protein that interact with HeLa cell expressed SARS-CoV-2) and anti ACE2 antibody (ACE2 Recombinant Rabbit Monoclonal Antibody (Cat# MA532307; interact with mouse ACE2) were obtained from Life Technologies Corporation.<br>Immunohistochemistry: The primary antibodies were mouse anti-NeuN (1:200, Cat# MAB377, Millipore Sigma, Billerica, MA, USA), anti-collagen IV (1:200, Cat# SAB4500369, Millipore) and anti-ACE2 antibody (1:100; Life Technologies. Cat# MA532307). The secondary antibodies were donkey anti-rabbit-Cy5 for collagen IV and ACE2 and donkey anti-mouse-Cy5 for the NeuN (Jackson ImmunoResearch, Inc., West Grove, PA. 1:500). We are now collecting lot numbers
Validation	The antibody against ACE2 was validated (see Suppl Figure 2a and b.

Animals and other organisms

Policy information about studies involving animals; ARRIVE guidelines recommended for reporting animal research

Laboratory animals	Mice: C57BL/6J (2-3 months old; Jackson Laboratory; Bar Harbor, ME, USA), male mice were used.
Wild animals	N/A
Field-collected samples	N/A
Ethics oversight	All animal studies were performed in accordance with the National Institute of Health guidelines and using protocols approved by the University Committee on Animal Resources.

Note that full information on the approval of the study protocol must also be provided in the manuscript.
